# Breastmilk from COVID-19 negative lactating mothers shows neutralizing activity against SARS-COV-2

**DOI:** 10.1038/s41598-023-42421-6

**Published:** 2023-09-19

**Authors:** Daniela Morniroli, Lucia Signorini, Maria Dolci, Giulia Vizzari, Andrea Ronchi, Carlo Pietrasanta, Lorenza Pugni, Fabio Mosca, Serena Delbue, Maria Lorella Gianni

**Affiliations:** 1 Department of Clinical Sciences and Community Health, University of Milan, Via Della Commenda 12, 20122 Milan, Italy; 2 Fondazione IRCCS Ca’ Granda Ospedale Maggiore Policlinico, NICU, Milan, Italy; 3 Department of Biomedical, Surgical and Dental Sciences, University of Milan, Milan, Italy

**Keywords:** Health care, Nutrition, Immunology

## Abstract

Breastmilk protects newborns from infections through specific and nonspecific compounds. This study investigated the neutralizing activity against SARS-CoV-2 of breastmilk from SARS-CoV-2 negative, unvaccinated mothers, and compared it to that from infected nursing mothers. We enrolled women after COVID-19 swab testing results upon maternity admission, and divided them into two groups: group A, COVID-19-positive mothers, and group B, negative mothers. Breastmilk was randomly sampled at 2, 7, and 20 days postpartum. We collected 19 samples for Group A and 41 for Group B. A microneutralization assay was used to determine the 50% neutralization (NT_50_) titre. The presence of neutralizing antibodies was also determined. Group A had 100% neutralizing samples at 2 days postpartum (T0), declining 7 days postpartum (T1) and 20 days postpartum (T2). Group B samples exhibited neutralizing activity mostly at 7 days postpartum (T1) (90%). Negative mothers' samples showed no correlation between NT_50_ titres and antibodies' presence, suggesting that non-specific breastmilk components may exert antiviral action against SARS-CoV-2.

## Introduction

The dissemination of new SARS-CoV-2 variants and their effects on the most fragile segments of the population such as nursing mothers and newborns are again raising interest in the possible protective effects against this virus.

At the beginning of the COVID-19 pandemic, the possibility of breastfeeding for mothers infected with SARS-CoV-2 was questioned by some scientific societies^1^. Almost three years after the beginning of the pandemic^2^, a large number of papers have been published in the literature showing that, where maternal conditions permit, breastfeeding is not only possible but should be encouraged^3^. In fact, in addition to the many benefits of breastmilk already known in the literature^4^, several studies have demonstrated the presence of antibodies against SARS-CoV-2 in breastmilk from both infected and vaccinated women^5–9^. A paper published by Collier et al. showed that both infection and vaccination elicited binding and neutralizing antibodies in breastmilk^10^.

However, the antiviral activity of breastmilk does not depend exclusively on specific antibodies directed against viruses previously encountered by the mother.

Some authors have speculated on a phylogenetic origin mainly for immune purposes of the mammary gland, as suggested by a correspondence between the antimicrobial substances of the mucous epithelial secretion and those secreted by lactocytes, described in a paper published by Vorbach et al.^11–14^.

It is now known that breastmilk contains numerous bioactive factors with proven non-specific antiviral capacity. These compounds, belonging to various classes of molecules such as lipids, enzymes and proteins, probably have a synergistic action whose mechanisms are not yet fully known^15^. This unspecific antiviral activity, independent from the presence of antigen-specific antibodies, is reasonably present in the breastmilk of all lactating women regardless of their infection status.

Given these premises, the aim of the study was to detect the presence and magnitude of neutralizing activity of breastmilk against SARS-CoV-2 from unvaccinated mothers who never experienced SARS-CoV-2 infection (COVID-19 negative, group B); and to compare it with breastmilk from SARS-CoV-2 infected lactating mothers (COVID-19 positive, group A).

## Methods

From November 2020 to May 2022, we screened mothers admitted to the maternity ward of a third-level care hospital and referral center for SARS-CoV-2 positive pregnant women. Before hospital admission for delivery, all mothers were tested for SARS-CoV-2 presence in a nasopharyngeal swab and subsequently referred to the well-baby delivery room and nursery or to the dedicated COVID-19 maternity ward. After enrollment, women were divided in two groups depending on their infectious status: group A, COVID-19 positive mothers, and group B, negative and unvaccinated mothers. Group B mothers declared to never had a previous positive swab and not had been vaccinated either by personal choice or because they were enrolled in the early months of the study (last trimester of 2020 and early 2021) when vaccination was not available for the general population. For group B mothers, having had a previous positive SARS-CoV-2 swab or COVID-19 infection at any time in their clinical history or having had even a single dose of SARS-CoV-2 vaccine were exclusion criteria. However, it should be considered that the mothers in group B could have had an asymptomatic infection previously, unknown to them and therefore not reported. Enrollment took place in the well-baby nursery and in the COVID maternity ward during the mother’s hospital stay (up to three days postpartum for negative mothers). The mean age of the mothers enrolled was 33.6 ± 5. Milk samples from mothers of both groups were randomly collected at various lactation stages: 2 days (T0—colostrum), 7 days (T1—transition milk) and 20 days (T2—mature milk) postpartum. Milk samples collected in a sterile test tube and obtained through hand expression at the end of a single session of breastfeeding or breast pumping. Written informed consent was obtained from all mothers enrolled prior to the milk sample collection. The study was approved by the Ethics Committee “Milano 2” of the Fondazione IRCCS Ca' Granda Ospedale Maggiore Policlinico and the study was conducted in accordance with the Declaration of Helsinki and the hospital’s and local relevant government guidelines and regulations. Milk samples were stored at − 80 °C after collection and then transferred at the Translational Research laboratory, fully equipped with a biosafety-level-3 (BSL3) facility.

### Microneutralization assay

For microneutralization assay a total of 15 000 VERO E6 cells (CRL-1586, American Type Culture Collection (ATCC) USA)/well were seeded in 96-well culture microplates and left to adhere overnight in complete medium at 37 °C, in a humidified atmosphere containing 5% CO_2_, until 80% confluence was reached. Milk samples were heat inactivated at 56 °C for 40 min and two-fold serially diluted in complete medium from 1:2 up to 1:1024 in quintuplicate. After dilution, a total of 200 TCID_50_ (50% Tissue Culture Infectious Dose) of SARS-CoV-2 virions (B.1 lineage) (SARS-CoV-2/human/ITA/Milan-UNIMI-1/2020, GenBank MT748758.1) were mixed with breastmilk dilutions and incubated for 1 h at 37 °C in a humidified atmosphere containing 5% CO_2_. After incubation, milk preparations were seeded on VERO E6 cell monolayer grown overnight on 96-well culture microplates and incubated for two hours at 37 °C in a humidified atmosphere containing 5% CO_2_. At the end, complete medium was added to each well and incubated again for 5 days at 37 °C in a humidified atmosphere containing 5% CO_2_. At day 5 post infection, each plate was inspected for cytopathic effects (CPE) with an inverted optical microscope. The 50% neutralizing endpoint titer (NT_50_) was determined by the means of the Reed and Muench method^16^. The test was performed in a biosafety level 3 (BSL3) facility.

### SARS CoV-2 NeutraLISA assay

The presence and magnitude of SARS-CoV-2 specific neutralizing antibodies in breastmilk samples was determined with the commercial kit SARS-CoV-2 ELISA assay (SARS CoV-2 NeutraLISA, Euroimmun, Italy), following the manufacturer’s instructions. Milk samples were centrifuged at 1500 rpm for 15 min. The creamy layer of fat globules was discarded to eliminate or reduce the inhibitory effects of fat on subsequent analyses. The milk whey supernatant was further centrifuged at 12,000 rpm for 10 min to remove creamy residues and the resulted milk serum was diluted 1:5 for the ELISA assay. Briefly, 100 uL of diluted samples were added to the microplate and incubated at 37 °C for one hour. Each well was then washed three times with washing buffer supplied by the kit, and 100 uL of enzyme conjugate was added to each well and incubated for 30 min at room temperature. After three washes, 100 uL of chromogen/substrate solution was added to each well and incubated for 15 min at room temperature, protect from light. The reaction was then stopped with 100 uL/well of stop solution and the plate was read at a wavelength of 450 nm. Blank, positive and negative control were added to each plate and each sample was tested in duplicate. The results were semi quantitatively expressed as percentage of inhibition (%IH) and analyzed as follow: %IH ≤ 20: negative, %IH ≥ 20 and < 35: borderline, %IH ≥ 35: positive. The %IH was calculated as follow:

$$100\%=\frac{Sample\,absorbance \times 100\%}{\mathrm{Blanck\,absorbance }}=\mathrm{ \%IH}.$$

### Statistical analysis

Stastistical analysis was performed with SPSS version 21 statistic software package (SPSS Inc., Chicago, IL, USA), while graph generation was performed using GraphPad Prism 9 for Windows (GraphPad Software, Boston, Massachusetts USA, www.graphpad.com). Categorical and numerical variables were described using medians and interquartile range (minimum/maximum values, IQR). Median NT_50_ values were compared using Mann–Whitney U test and Kruskal–Wallis nonparametric test as appropriate. Pearson’s correlation analysis was used to analyze correlation between %IH and paired NT_50_ values.

## Results

### Breastmilk NT_50_ titers

We collected a total of 60 breastmilk samples form 49 women enrolled. Fifteen SARS-CoV-2 positive mothers (Group A), and 34 SARS-CoV-2 negative mothers (Group B) A total of 19 and 41 milk samples were collected in group A and B respectively. In group A, the number of breastmilk samples for each time point was as follow: 5 samples at T0, 7 samples at T1 and T2 (Table 1).

**Table 1:** Breastmilk samples NT_50_ values (SARS-CoV-2 B.1 lineage).

	Group A (n = 19)			Group B (n = 41)			
	T0 (n = 5)	T1 (n = 7)	T2 (n = 7)		T0 (n = 24)	T1 (n = 10)	T2 (n = 7)
Median	1:32.0	1:22.8	1:3.2		1:11.3	1:4.0	1:2.8
Min	1:16.0	1:4.0	1:2.3		1:2.8	1:2.3	1:2.3
Max	1:512.0	1:256.0	1:20.7		1:81.6	1:6.3	1:6.3

*n* number of milk samples.

**Figure 1:**
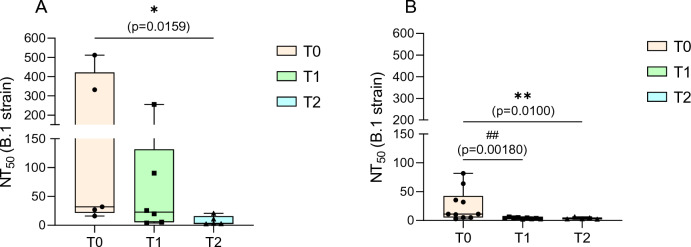
NT_50_ values against SARS-CoV-2 B.1 lineage in group A (**A**) and group B (**B**) in breastmilk samples at each timepoint. The boxes extend from the 25th to 75th percentiles, plots whiskers down to the minimum and up to the maximum value, each individual value is a point superimposed on the graph and the middle lines of each box shows medians. *p = 0.0159 (**A**), ^##^p = 0.00180, **p = 0.0100 (**B**) (statistical test: Mann–Whitney U test).

**Figure 2:**
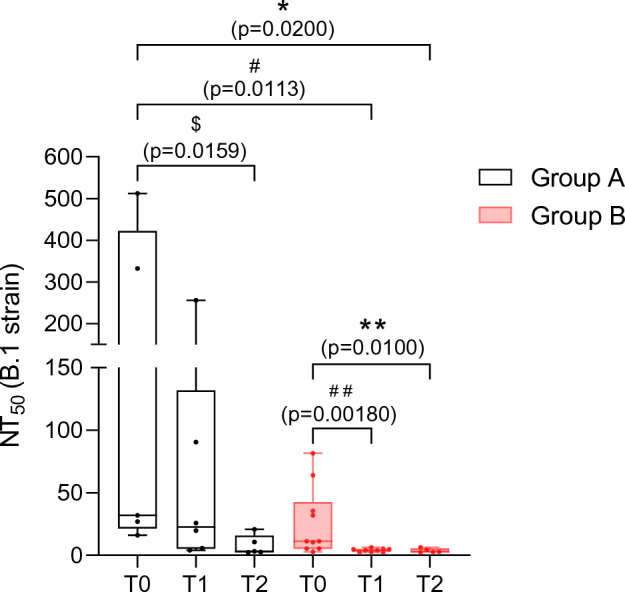
NT_50_ values against SARS-CoV-2 B.1 lineage in group A (white boxes) and group B (red boxes) in breastmilk samples at each timepoint. The boxes extend from the 25th to 75th percentiles, plots whiskers down to the minimum and up to the maximum value, each individual value is a point superimposed on the graph and the middle lines of each box shows medians. ^$^p = 0.0159, ^##^p = 0.00180, **p = 0.0100 (statistical test: Mann–Whitney U test), *p = 0.0200, ^#^p = 0.0113 (statistical test: Kruskal–Wallis nonparametric test).

In group B, the number of breastmilk samples for each time point was as follow: 24 samples at T0, 10 samples at T1, 7 samples at T2 (Table 1). Median NT_50_ values in each group for each timepoint are detailed in Table 1 and Figs. 1 and 2.

A total of 16/23 (84.21%) and 24/41 (58.54%) breastmilk samples showed neutralizing activity against SARS-CoV-2 B.1 lineage in groups A and B respectively. Time point analysis showed a total of 5/5 samples (100.00%) at T0, 6/7 samples (85.71%) at T1 and 5/7 samples (71.43%) at T2 with neutralizing activity in group A and 10/24 (41.67%) at T0, 9/10 (90.00%) at T1 and 5/7 (71.43%) at T2 with neutralizing activity in group B. Median NT_50_ values were 1:32.00 at T0, 1:22.76 at T1, and 1:3.21 at T2 in Group A and 1:11.31 at T0, 1:4.00 at T1 and 1:2.83 at T2 in Group B (Table 1). Significant differences were found in median NT_50_ values between T0 and T2 (p = 0.0159) in group A (Fig. 1A) and between T0 and T1 (p = 0.0018) and T0 and T2 (p = 0.0100) in group B (Fig. 1B). Comparison between median NT_50_ values between group A and B showed a significant difference between T0 (group A) and T1 (group B) (p = 0.0113) and T0 (group A) and T2 (group B) (p = 0.0200) (Fig. 2).

### SARSCoV-2 NeutraLISA assay

To detect the presence of SARS-CoV-2 specific neutralizing antibodies in milk samples a specific SARS-CoV-2 semiquantitative ELISA assay was performed. Breastmilk %IH was calculated for all the enrolled women, during the different time points. The analysis was not possible for 2 samples at T1 in group A, 7 samples at T0, 6 samples at T1 and 4 samples at T2 in group B, due to the low amount of collected breastmilk. In group A, the number of analyzed breastmilk samples for each time point was as follow: 5 samples at T0, 5 samples at T1 and 7 samples at T2 (Table 2).

**Table 2:** Breastmilk %IH values.

	Group A (n = 17)			Group B (n = 24)			
	T0 (n = 5)	T1 (n = 5)	T2 (n = 7)		T0 (n = 17)	T1 (n = 4)	T2 (n = 3)
Median	43.4	38.8	35.3		31.2	40.2	23.1
Min	29.1	26.8	21.6		1.8	28.3	13.8
Max	85.7	96.1	44.1		68.1	61.8	49.2

Interpretation of the semiquantitative result: %IH ≤ 20: negative, %IH ≥ 20 and < 35: borderline, %IH ≥ 35: positive.

*n* number of milk samples.

**Figure 3:**
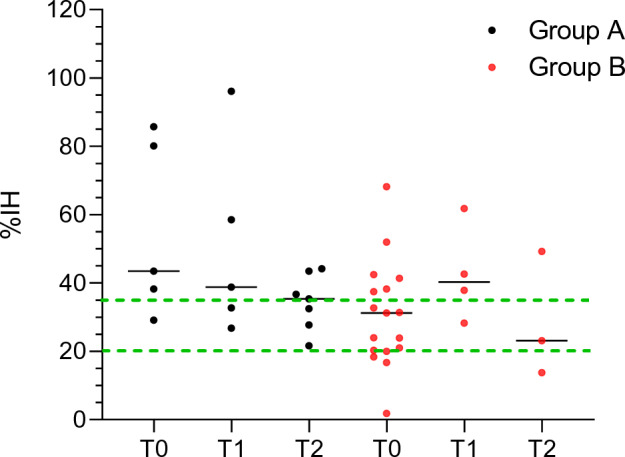
%IH values against SARS-CoV-2 in group A (dark dots) and B (red dots) in breastmilk samples at each timepoint. The black lines represent the median %IH values. The green dotted lines represent the 20%IH and 35%IH. Interpretation of the semiquantitative result: %IH ≤ 20: negative, %IH ≥ 20 and < 35: borderline, %IH ≥ 35: positive.

Regarding group B, the number of analyzed breastmilk samples for each time point was as follow: 17 samples at T0, 4 samples at T1, 3 samples at T2 (Table 2). A total of 13/17 (76.5%) and 10/24 (41.7%) breastmilk samples showed presence of SARS-CoV-2 specific neutralizing antibodies in groups A and B respectively. Time point analysis showed a total of 4/5 samples (80.0%) at T0, 3/5 samples (60.0%) at T1 and 4/7 samples (57.1%) at T2 with %IH ≥ 35 in group A and 6/17 (35.3%) at T0, 3/4 (75.0%) at T1 and 1/3 (33.3%) at T2 with %IH ≥ 35 in group B (Fig. 3).

**Figure 4:**
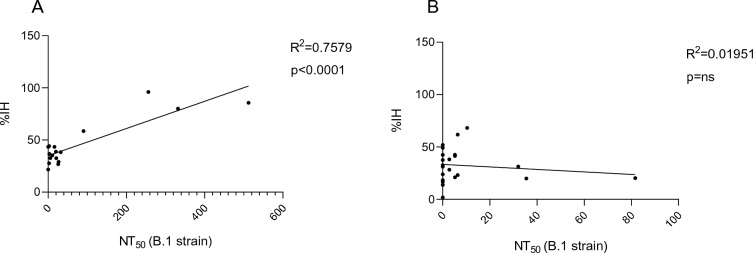
Pearson’s correlation between breastmilk %IH and NT_50_ titres. Pearson’s correlation analysis showed significant positive correlation in group A (R^2^ = 0.7579, p < 0.0001) (**A**) and no correlation in group B (R^2^ = 0.01951, p = ns) (**B**).

Pearson’s correlation analysis showed significant positive correlation between %IH and paired NT_50_ for group A (R^2^ = 0.7, p < 0,0001) and no correlation in group B (R^2^ = 0.01951, p = ns) (Fig. 4).

## Discussion

Remarkably, the samples from the negative moms exhibited neutralising potency, with the majority of samples with this antiviral impact being from the second time point (41% at T0 versus 90% at T1). In the B group of negative mothers, there was a statistically significant difference between the endpoint colostrum titre (NT50 1:11.3) and the titres seen at later time points, as determined by calculating NT50 (1:40 at T1 and 1: 2.8 at T2). The results of this study confirm the ability of milk from COVID-positive women to neutralize SARS-CoV-2. This confirms what already found in the literature^17^ although in our case the percentage of neutralizing samples at T0 was 100% and then decreased at the other time points (85% at T1 and 71% at T2). The neutralizing potency expressed by NT_50_ in group A also shows a statistically significant progressive reduction from T0 to T2. This could be due to the fact that the mothers' COVID positivity was established at the time of delivery and therefore temporally at the time of colostrum sample collection (T0) with a possible progressive reduction of antibody levels in milk with time after infection, although previous studies in the literature have shown persistence of antibodies in breastmilk even at 90 days^18^ and up to 6 months^19^. Colostrum collection (2 days postpartum-T0) was very difficult for mothers and sometimes impossible despite their best effort. As a result, the number of samples in the two groups at this time points differs, with 5 samples in Group A and 17 in Group B at T0, colostrum collection. Taking into account the significant efforts made by mothers, every hardly obtained sample was analyzed and presented in this article.

With regard to the presence of anti-SARS-CoV-2 antibodies by ELISA assay, as expected, the positive mothers in group A showed a variable presence and amount of antibodies directed against the virus at the various time-points. Again, this presence tends to decrease over time although not significantly, as shown in previous studies^20^.

Surprisingly, the presence of antibodies directed against SARS-CoV-2 has also been demonstrated in the milk of negative mothers (group B), but to a lesser extent and with a lower IH% than the milks of mothers in group A. This result could be due to any previous SARS-CoV-2 infections, which were asymptomatic and therefore unrecognized and unreported by the mother or, intriguingly, to the presence of antibodies, especially of the IgA class, directed against other Coronaviruses, but capable of cross-reacting with SARS-CoV-2, and thus affecting the test, as already shown by other previous studies in the literature on plasma and saliva^21,22^.

The specific activity against SARS-CoV-2 of the antibodies detected in the milk of positive mothers is also supported by the direct correlation found between the presence of the antibodies and the neutralizing activity of the breastmilk samples. On the contrary, this correlation was not found for the milks of negative mothers. This observation supports the hypothesis that the antiviral activity might be exerted by non-specific components naturally present in the breastmilk, and independently by the activity of antibodies. It is indeed already known in the literature that breastmilk, and especially colostrum, is rich in anti-infective substances with nonspecific activity. Several compounds in breastmilk with proven antiviral capacity have already been described in the literature.

Among the antiviral components of colostrum, cytokines, polyunsaturated fatty acids, immuno-stimulating proteins, glycoproteins such as lactoferrin (LF), glycated components such as mucins, human milk oligosaccharides (HMOs), and extracellular vesicles have known broad antimicrobial activity^23^. Greater understanding of these bioactive compounds may aid in the development of new strategies to combat viral infections, even in old age.

The American Academy of Pediatrics (AAP)^24^ confirmed that breastfeeding could help protect neonates against viral infection through the active mediators of human milk^25^.

Functional proteins in breastmilk have aroused interest as factors involved in its antiviral activity^26^. Among these, tenascin-C is a well-studied protein for its ability to bind to the chemokine co-receptor site of HIV (Human Immunodeficiency Virus), possibly explaining why most HIV-1-exposed breastfed infants are protected against mucosal HIV-1 transmission^27^.

It has been observed that the immune response is necessary for viral infection inhibition; this mechanism is mediated by genes that encode active mediators, such as LF^28^. Reghunathan et al.^29^ discovered elevated levels of LF during SARS-CoV-2 infection, which enhanced the immune response via Natural Killer cell activation. LF inhibits CoV-host cell binding by blocking the interaction between CoV and Heparan sulfate proteoglycans (HSPGs)^30^. Furthermore, it prevents virus spike protein binding to ACE-2, preventing virus attachment and fusion as well as host cells^31^. LF may also interfere with CoV-2 HSPGs and ACE-2 pathways. Lang and colleagues^28^ discovered that LF can prevent SARS pseudovirus entrance into host cells. It is known that virus adherence to the host cell is required for infection, and CoV-2 uses glycoprotein and ACE-2 metallopeptidase receptors; in the presence of receptor analogs, these mediators and the virus will compete. Among many others, a study by Kell and colleagues described how lactoferrin could interfere with SARS-CoV-2 entry into the cell by binding to heparan sulphate molecules that serve as the virus' first contact with the cell^32^.

Oligosaccharides are another class of molecules in breastmilk that show promise for their antiviral capabilities. These complex combinations of glucose, galactose, fucose, N-acetylglucosamine and N-acetylneuraminic acid have already been shown to have antiviral capacity. They may have the same effect on SARS-CoV-2 by acting as a decoy and occupying or modifying virus receptor sites, preventing entry into the cell.

HMOs may interfere with virus binding, lowering their pathogenicity. In addition, Pandey et al.^33^ found that HMOs have antiviral efficacy against a wide variety of avian influenza viruses and may limit pathogen attachment to host cells in the same manner that human milk mediators may inhibit CoV-2 adherence to host cells.

Interestingly, not all nursing mothers produce the same classes of oligosaccharides. Their synthesis and composition from crucial components are dependent on their fucosylation by the fucosyltransferases FUT2 (Secretor gene) and FUT3 (Lewis gene), which, like the blood group antigens FUT2 and FUT3 dependent, vary between mothers^34^. This inter-individual variability within the pool of oligosaccharides may underlie the significant variability in neutralising activity demonstrated by the colostrum samples from the negative mothers in our sample.

This exploratory pilot investigation is unquestionably constrained by its small sample size and the absence of a comparative spectrometric examination between samples to determine the aspecific compounds that could explain these outcomes.

To the best of our knowledge, it is the first study to assess the extent of the antiviral activity of breastmilk collected from negative, unvaccinated mothers and therefore not only suggesting but measuring a possible role for the innate human milk compounds against SARS-CoV-2.

This study provides many fascinating insights for future research, such as identifying the differences in composition between neutralising and non-neutralising samples and therefore begin to trace a path for understanding which substances in breastmilk have this unspecific antiviral effect and what could be the clinical implications of their supplementation for human health.

## Data availablity

The datasets generated and analysed during the current study are not publicly available due to local privacy regulations but are available from the corresponding author upon reasonable request.
